# Impact of immobilization strategies on the activity and recyclability of lipases in nanomagnetic supports

**DOI:** 10.1038/s41598-022-10721-y

**Published:** 2022-04-26

**Authors:** Thais de Andrade Silva, Wanderson Juvêncio Keijok, Marco Cesar Cunegundes Guimarães, Sérvio Túlio Alves Cassini, Jairo Pinto de Oliveira

**Affiliations:** 1 Federal University of Espírito Santo, Av Marechal Campos 1468, Vitória, ES 29040-090 Brazil; 2 Federal University of Espírito Santo, Av Fernando Ferrari 514, Vitória, ES 29075-910 Brazil

**Keywords:** Biocatalysis, Enzymes, Nanoscience and technology

## Abstract

The use of enzymes immobilized on nanomagnetic supports has produced surprising results in catalysis, mainly due to the increase in surface area and the potential for recovery and reuse. However, the meticulous control of the process and difficulties in reproducibility have made industrial-scale applications unfeasible. Furthermore, the role of conjugation strategies in the catalytic activity and recycling of catalysts is unclear. Therefore, the objective of this study was to compare the conjugation of enzymes on nanomagnetic supports through physical adsorption (naked) or covalent bonding with mercaptopropyltrimethoxysilane (MPTS) and aminopropyltriethoxysilane (APTS) ligands. The free lipase obtained from *Rhizomucor miehei* was used as a model enzyme. Total protein and enzyme activity were determined using spectrophotometry (UV–Vis) and the p-nitrophenyl palmitate (p-NPP) hydrolysis method. The results indicated that a more significant enzyme surface loading does not always mean better immobilization success. The physical adsorption binding strategy had higher surface loading and low catalytic activity. On the other hand, covalent coupling with free NH2 had an excellent catalytic activity with very low surface loading. Finally, we show that recyclability can be improved with conjugation mediated by disulfide bonds. The findings presented here are essential for developing nanoconjugates with high enzymatic activity, which can guarantee the success of several industrial applications.

## Introduction

Lipases stand out among enzymes because they catalyze reactions and synthesis in chemo-, regio-, and enantioselective manners. For example, these enzymes hydrolyze triglycerides at the water–oil interface, releasing fatty acids and glycerol^1^. In addition, lipases can catalyze synthesis reactions, such as transesterification, esterification, and interesterification in non-aqueous media^2^. Such versatility makes lipases extremely interesting and recognized as the biocatalysts of the future^3^. However, one of the problems of using enzymes as homogeneous catalysts is their recovery. Thus, it is necessary to use supports that retain the enzyme, allowing its recovery by maintaining its catalytic characteristics, thus increasing the efficiency of the reaction.

In recent years, several immobilizing supports have been developed^4–9^. Enzymatic immobilization has been suggested as an alternative to reduce the limitations of soluble enzymes, increasing their stability and facilitating recovery and reuse. This allows material and energy savings in the biocatalytic process^10^. Special attention has been given to magnetic nanoparticles (MNPs) as an alternative to conventional supports^11–26^. MNPs add new properties to immobilizing supports, such as high surface area, greater temperature tolerance, good chemical reactivity, and strong interactions with enzymes^27–29^. Furthermore, the characteristic magnetic field of these nanoparticles enables an efficient recovery of the enzyme complex, thus preventing contamination of the final reaction product. In addition, nanoscale supports maximize enzyme stability, modulating catalytic specificity and displaying low resistance to mass transfer, thus improving diffusion and reducing operational cost^30^.

The nano-bio interface comprises the dynamic, physicochemical, kinetic, and thermodynamic interactions between the surfaces of nanomaterials and enzymes. Strategies used for immobilization include physical adsorption and covalent coupling. Physical adsorption is the easiest and most used method, but it often suffers from random orientation and denaturation of bound proteins, giving rise to poor reproducibility^31^. On the other hand, covalent bonding promotes more stable immobilization of lipases and better reproducibility. However, it has a lower yield and can also cause disordered orientation, resulting in loss of biological activity^32^. An increase in catalytic activity by a factor of 2 or more has already been demonstrated by covalent coupling^33^ and recovery rates of up to 10 cycles have been reported for this strategy^16,34^.

Several recent studies have explored conjugation strategies that use physical adsorption^12,20,26,33,35^ and covalent coupling^13,14,17–19,21,25,36–38^. These strategies further the development of biocatalysts with good chemical stability, magnetic recovery, and high recyclability. However, there is a considerable gap regarding the influence of the ligand beyond the immobilization yield, as in the catalytic activity, in the catalyst recovery rate, and the chemical stability in relation to the use of free lipases. One of the main reasons for this gap is that the usual conjugation methods are random and untargeted. Furthermore, in many cases, the enzyme's active site is involved in the interface with the metal, decreasing the efficiency of the immobilized catalyst. The correct understanding of these mechanisms can maximize the success of several applications involving biocatalysts in nanomagnetic supports.

This study evaluated the effects of conjugation methods on the catalytic activity and recyclability of lipases on magnetic platforms. Our main objective was to maximize the success of applications involving lipases immobilized on magnetic nanoparticles and support the development of versatile and reproducible platforms. The methods of physical adsorption, covalent coupling with MPTS ligand (SH), and covalent coupling with APTS ligand (NH_2_) were studied. Immobilization efficiency was determined through the quantification of enzymes on the metal surface. Catalytic activity was determined through the para nitrophenol-palmitate hydrolysis method. Recyclability rate was evaluated during five reuse cycles due to lipase activity. In addition, nanoconjugates were investigated by TEM, XRD, Raman, FTIR, Zeta Potential, and DLS.

## Methods

### Materials

FeCl_2_·4H_2_O (Sigma-Aldrich 44939), FeCl_3_·6H_2_O (Sigma-Aldrich F2877), Ammonium Hydroxide (Prochemios), Sodium Citrate (Dynamica 1146), (3-Mercaptopropyl)Trimethoxysilane (Sigma-Aldrich 175617) (MPTS), 3-Aminopropyltriethoxysilane (Sigma-Aldrich 440140) (APTS), Lipase from *Rhizomucor miehei* (Sigma-Aldrich L4277), p-Nitrophenyl palmitate (Sigma-Aldrich N2752) (p-NPP), Isopropyl Alcohol (Dinâmica, Brazil), Ethyl Alcohol (Exodus, Brazil), Phosphate-Saline Buffer (Sigma-Aldrich P4417), Argon gas (Oxivit, 99.999%). Ultrapure Water (Millipore Synergy Merck), Neodymium magnet 50 × 50 × 12 mm (Supermagnet, Brazil). In addition, all glassware was sanitized with aqua regia (HCl: HNO_3_) and rinsed ten times with ultrapure water before the experiments.

### Synthesis and functionalization of magnetic nanoparticles

MNPs were synthesized by the chemical coprecipitation method with adaptations from Hongjian et al.^39^. Briefly, 40 mL of 0.09 M FeCl_2_·4H_2_O and 40 mL of 0.18 M FeCl_3_·6H_2_O were mixed in a flask. After complete dissolution, 7.5 mL of 28% NH_4_OH was added at a rate of 5 mL min and the mixture kept under constant stirring for 10 min at 65 °C, generating a black precipitate of Fe_3_O_4_. Soon after, 5.5 g of sodium citrate was added to the reaction, which was kept under constant stirring for further 30 min. Finally, the reaction was stopped by chilling in an ice bath.

Surface functionalization of the MNPs was performed through the covalent coupling method using the APTS and MPTS ligands. From a 20 mL aliquot of the MNPs suspension, the Fe_3_O_4_ precipitate was separated by removing the supernatant using magnetism. The pellet was resuspended in 20 mL of a 0.043 M APTS and 0.054 M MPTS alcoholic solution and kept under constant agitation at 150 rpm for 40 h at 28 °C. The particles obtained were black and exhibited a strong magnetic response. The material was washed three times with ethanol and distilled water, and the resulting precipitates were oven-dried at 60 °C and stored for future use.

### Characterization of nanomaterials

The morphology and distribution of iron MNPs were analyzed under a Transmission Electron Microscope JEM-1400, JEOL, USA inc., operated at 120 kV with a tungsten filament. X-ray diffractometry was performed with scanning in the 2θ region, from 30° to 90°, at 0.01° per minute, with a time constant of 2 s, using a Phillips PW 1710 diffractometer (Cu ka radiation). The vibrating sample magnetometer (VSM) data were measured by MPMS SQUID 7.0. Raman spectroscopy analysis was performed with the Optosky Handheld RS4 equipment with a spectral range from 400 to 2300 cm^−1^ using a 785 nm laser. Infrared Spectroscopy measurements were performed using Agilent Cary 630 FTIR equipment. UV–Vis absorption readings were taken on a scale from 200 to 800 nm (Ocean Optics UBS 2000 spectrophotometer), and ultrapure water was used to perform the blank reading. Data were processed in the OriginPro 8.5 SR1 software. Dynamic Light Scattering (DLS) and Zeta Potential (PZ) measurements were taken with a Litesizer 500 equipment (Anton Paar), using 2 mL of colloidal sample. DLS final values were expressed in nm and the zeta potential in mV.

### Conjugation of lipases with magnetic nanoparticles

For conjugation, the commercial lipase from *Rhizomucor miehei* was used as an enzyme model. Functionalized MNPs were washed once with ethanol and three times with distilled water to remove excess ligands. Conjugation was performed with 30 mg mL^−1^ lipase in 10 mM PBS buffer, pH 7.2, using glutaraldehyde for covalent coupling assays as an activation reagent. The reaction took place under stirring at 150 rpm for 1 h at room temperature. The conjugation yield was calculated indirectly by the amount of free enzyme in the supernatant after washing with PBS buffer using an external magnetic field. Lipases were quantified by UV–Vis Spectrophotometry from a standard curve of enzyme concentration, considering the absorption peak at 250 nm characteristic of the enzyme. Confirmation of lipase conjugation on the metal surface was also qualitatively evaluated by FTIR and Raman. All data were made available in the supplementary material (Fig. S1, Tables S1 and S4).

### Enzyme activity

The enzymatic activity of the immobilized lipase was quantified by UV–VIS spectrophotometry using the p-NPP hydrolysis method (maximum abs 410 nm). For this assay, 0.5 mL of the nanoconjugates were separated by a magnetic field for 5 min. The pellet was resuspended in 1 mL of the reaction medium, composed of 35 μL of 15 mM p-NPP in isopropyl alcohol and 965 μL of PBS buffer (100 mM, pH 7.2). The reaction took place under light stirring for 5 min at 25 °C. Enzyme activity was determined in U mg^−1^, in which U (unit of enzyme activity) is given in µmol/g min^−1^ per mg of lipase. U was calculated using Eq. (1).

1 $$ {\text{A}} = \, \varepsilon \cdot {\text{bc}} $$

where A is the absorbance, ε the molar extinction coefficient of p-NPP (1.50 × 10^–4^ mol L/cm min), b is the optical path (1 cm), and c is the molar concentration of p-NPP. All data were made available in the supplementary material (Fig. S1, Tables S2–S4).

### Stability assessment (pH and temperature)

To evaluate the stability, the immobilized enzymes were incubated in the reaction mixture at different pH values (5–9) using 10 mM acetate buffer (pH = 5), 10 mM phosphate buffer (pH = 6–7) and Tris–10 mM HCl (pH = 8–9) for 5 min at 25 °C. Catalytic activity was evaluated as described in section “Enzyme activity”. The optimal temperature for immobilized enzyme activity was determined by incubating the reaction mixture for 5 min in 10 mM Tris–HCl buffer (pH = 8) at temperatures ranging from 25 to 60 °C. Catalytic activity was evaluated as described in Sect. Enzyme activity. All data were made available in the supplementary material (Tables S5, S6).

### Residual activity (recyclability)

The enzymatic activity of the conjugates was analyzed after five washing cycles and separated by an external magnetic field for 5 min. A washing step with PBS buffer (100 mM, pH 7.2) was performed after each cycle to remove the reaction medium from the previous cycle. Residual activity was assessed according to the protocol described in the topic 2.5. All data were made available in the supplementary material (Table S7).

## Results and discussion

### Synthesis and characterization of magnetic nanoparticles

**Figure 1:**
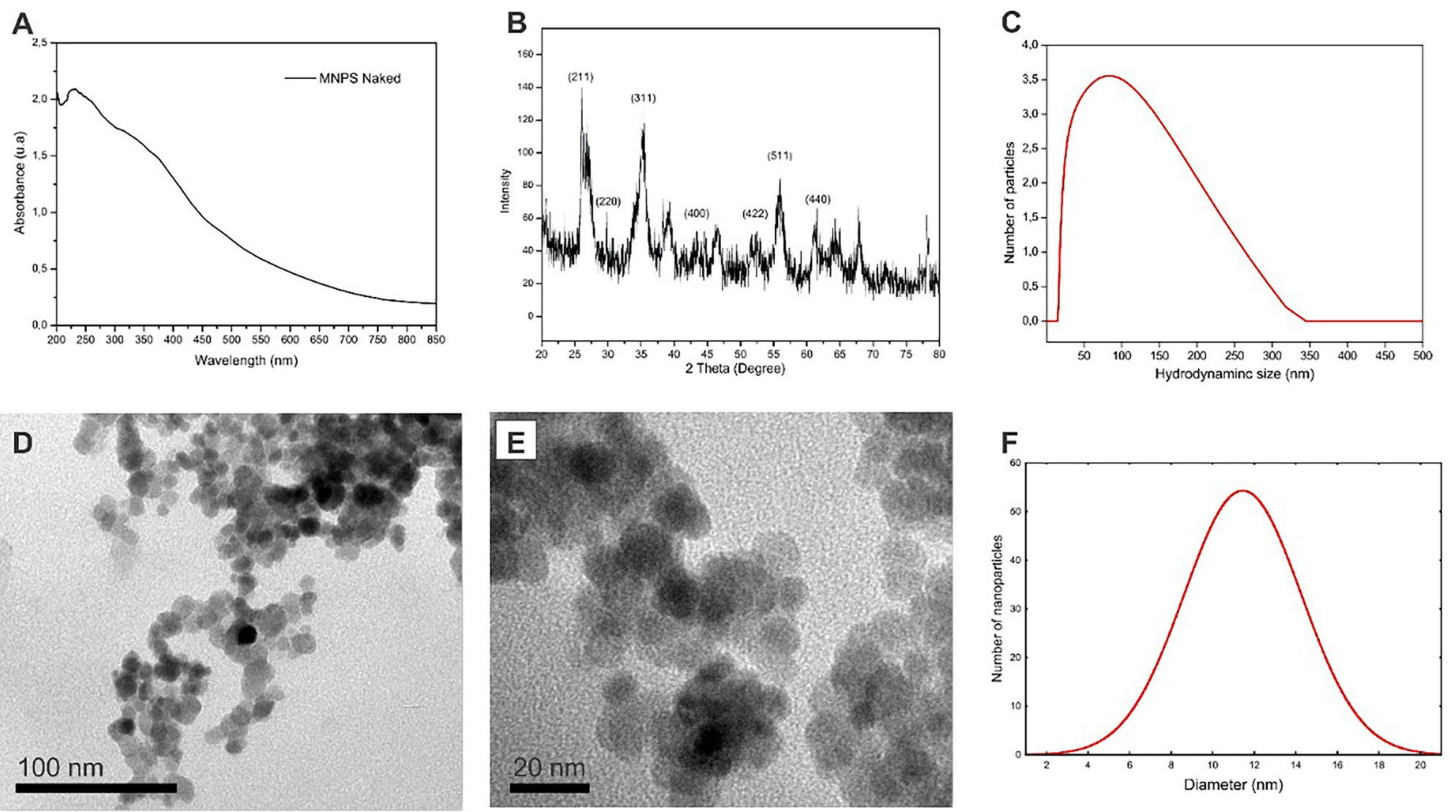
UV–Vis spectrum (**A**) and XRD of Fe_3_O_4_ particles (**B**). Histogram obtained by DLS (**C**). Images of Fe_3_O_4_ MNPs by TEM (**D**,**E**). Histogram of the size distribution of MNPs obtained by TEM. (**F**).

Magnetic nanomaterials were synthesized by chemical coprecipitation of Fe^2+^/Fe^3+^ ions in alkaline solution under heating, in an inert argon atmosphere. A black precipitate was observed after synthesis, and the material showed high magnetization when the magnetic field was applied. The UV–VIS spectrum (Fig. 1A) revealed the formation of nanomaterials by a color change and light absorption across the entire spectrum evaluated (200–850 nm). A characteristic absorption band in the ultraviolet region is due to the surface plasmon resonance of iron.

The patterns observed by XRD analysis (Fig. 1B) revealed that the crystalline planes are characteristic of iron nanoparticles and that the predominant crystallographic orientation is that of spinel^40–42^. Combining the peaks with the JCPDS file also indicated that the crystallographic system has a cubic structure. Analysis of the images by TEM (Fig. 1D–F) showed that the MNPs had a relatively spherical shape, an average size of 10–12 nm, and good mono dispersion (Fig. 1C). The DLS experiment showed that hydrated MNPs had an average size of 100 nm. The hydrodynamic size is generally more significant than the actual size of MNPs because of extra hydrated layers adhered to the surface. The zeta potential was − 75 mV, indicating a high degree of stability, possibly because OH groups adhere to the metallic surface. The magnetic hysteresis curve (Fig. S2) of nanomagnetic supports has an excellent magnetic property, with a saturated magnetization value of 73.20 emu g^−1^.

### Functionalization of MNPs with APTS and MPTS ligands

Surface modification of iron magnetic nanoparticles is essential for enzyme immobilization and the gain of colloidal stability. Strategies using silane groups have been preferentially explored. These ligands prevent iron oxidation, preserve the magnetization of nanomaterials, and allow conjugation with various functional groups^43–45^. The formation of a monolayer using the ligands APTS and MPTS facilitates the immobilization of enzymes through the available organic groups (NH_2_ and SH).

**Figure 2:**
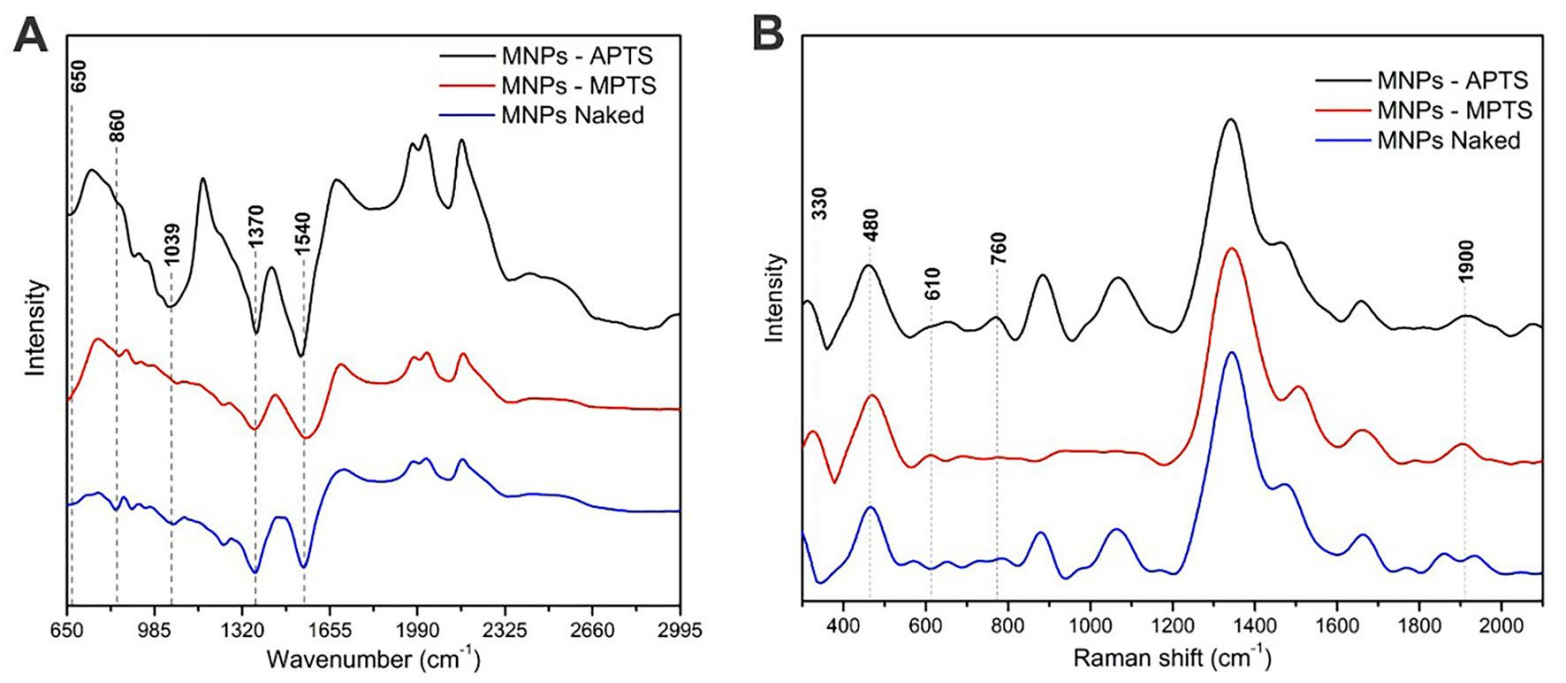
FTIR (**A**) and Raman (**B**) spectra of naked Fe_3_O_4_ MNPs and functionalized with MPTS and APTS.

Surface modification was performed by adding primary amino groups (APTS) and free thiol groups (SH) in ethanolic solution. Bonding to the metallic surface was made possible by the strong interaction between the silane groups and the metallic surface of the iron. Functionalization was confirmed by FTIR analysis (Fig. 2A) and Raman spectroscopy (Fig. 2B). In addition, non-functionalized magnetic nanoparticles (naked) were also studied for conjugation by physical adsorption with enzymes.

The FTIR analysis confirmed the silane coverage using the APTS and MPTS ligands on the surface of MNPs. The initial low-intensity band around 650 cm^−1^ is characteristic of Fe–O vibrations, indicating that the synthesized materials consist of Fe_3_O_4_ nanoparticles, also evidenced by XRD. Furthermore, in this region, the band is more prominent for MNPs-APTS and MNPs-MPTS, suggesting a Fe–O–Si stretching vibration overlapping the Fe–O vibrations. The absorption peaks at 1370 and 1540 cm^−1^ can be attributed to the structural groups NH and COOH, possibly present in the sample due to the synthesis process using ammonium hydroxide and sodium citrate. The Si–O stretching vibrations at 1039 cm^−1^ observed in MNPs-APTS and Si–O–H bending vibrations at 860 cm^−1^ observed in MNPs-APTS and MNPs-MPTS confirm the silane coverage and the successful functionalization^46,47^.

Functionalization was also confirmed by Raman spectroscopy (Fig. 2B). The peaks at 330 and 480 cm^−1^ present in all spectra correspond to Fe–O vibrations^48^. A low-intensity band at 610 cm^−1^ in the MNPs-MPTS is characteristic of SiO_2_ vibrations. The weak S–H stretching band appears near 2500 cm^−1^^49^.The appearance of a shoulder at 760 cm^−1^ in the MNPs-APTS spectrum is related to Si–O–Si vibrations and confirmed the presence of APTS^50^. The peak at 1900 cm^−1^, attributed to the C–C stretching vibrations in the APTS and MPTS ligands, also confirm functionalization^51^.

### Efficiency of the immobilization of lipases on magnetic supports

**Figure 3:**
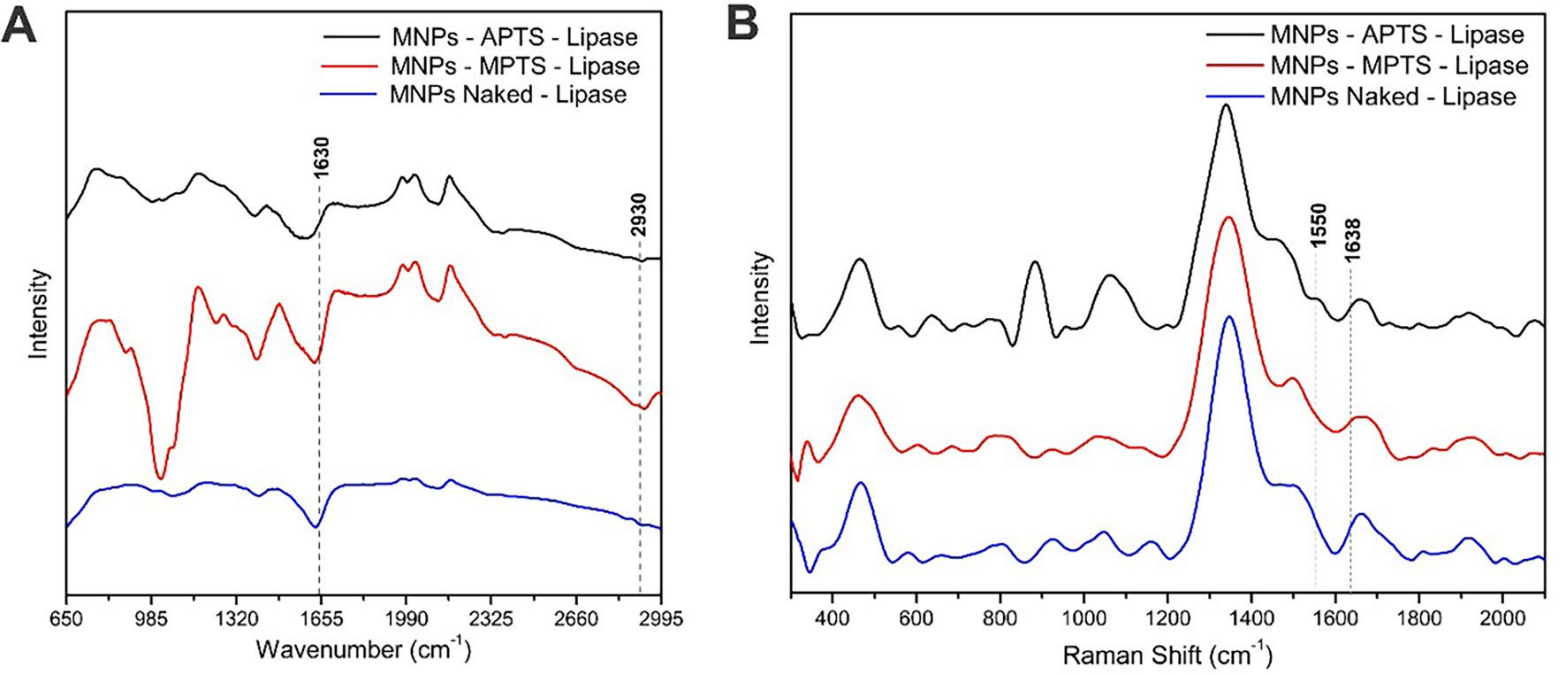
FTIR (**A**) and Raman (**B**) spectra of lipase-conjugated Fe_3_O_4_ MNPs.

The lipase of *Rhizomucor miehei* was used as a model enzyme. Conjugation was evaluated by physical adsorption (MNPs-naked) and covalent coupling (MNPs-APTS and MNPs-MPTS). FTIR and Raman spectra were also recorded after immobilization of the enzymes. In all samples, the FTIR spectra (Fig. 3A) showed a peak near at 1630 cm^−1^, which is characteristic of type 1 amide and type 2^52^. Primary amides (–CO–NH–) exhibit C=O stretching at 1680–1660 cm^−1^ (referred to as the amide I band) and NH2 bending at 1650–1620 cm^−1^ (referred to as the amide II band)^49^. C=N stretching also occurs in this region and is usually stronger.

This peak was more pronounced in MNP-APTS and MNP-MPTS, possibly due to covalent anchoring. The peaks at 2930 cm^−1^ originated from the vibrations caused by the elongation of C–H bonds of the alkyl chains present in the structure of enzymes^53^. In the Raman scattering spectra of the lipase-conjugated MNPs (Fig. 3B), it is possible to notice the amide I and amide II bands at 1638 and 1550 cm^−1^, respectively^54^.

**Figure 4:**
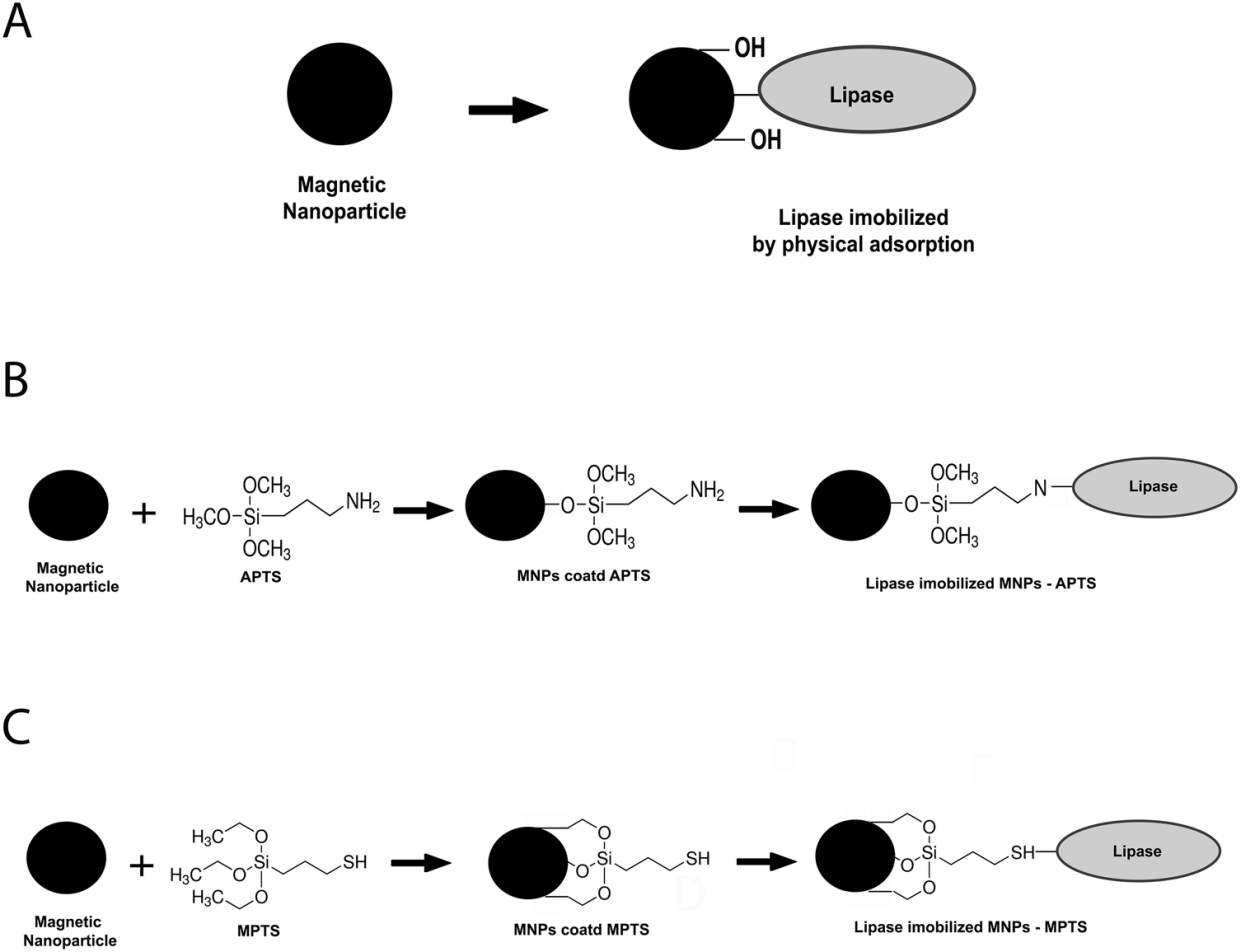
Schematic representation of immobilization of lipases by physical adsorption (**A**), immobilization by covalent coupling using the APTS ligand (**B**), and immobilization by covalent coupling using the MPTS ligand (**C**).

Proteins immobilized on the metallic surface were quantified through a standard curve by spectrophotometry (Fig. S1A,B). Lipase activity was determined through the p-NPP hydrolysis method. Catalytic efficiency tests were carried out at 25 °C for 5 min, according to previous optimization (Fig. S1C,D). The quantification of immobilized lipase was expressed as milligrams of enzyme per gram of nanomagnetic support. The schematic representation of immobilization of lipases by physical adsorption and covalent coupling using the APTS/MPTS ligands is shown in Fig. 4. Confirmation of the covalent bond as evidenced by the desorption study, where the nanoconjugates were placed in contact with a strong electrolyte solution. Details can be seen in Fig. S3 of the supplementary material.

**Figure 5:**
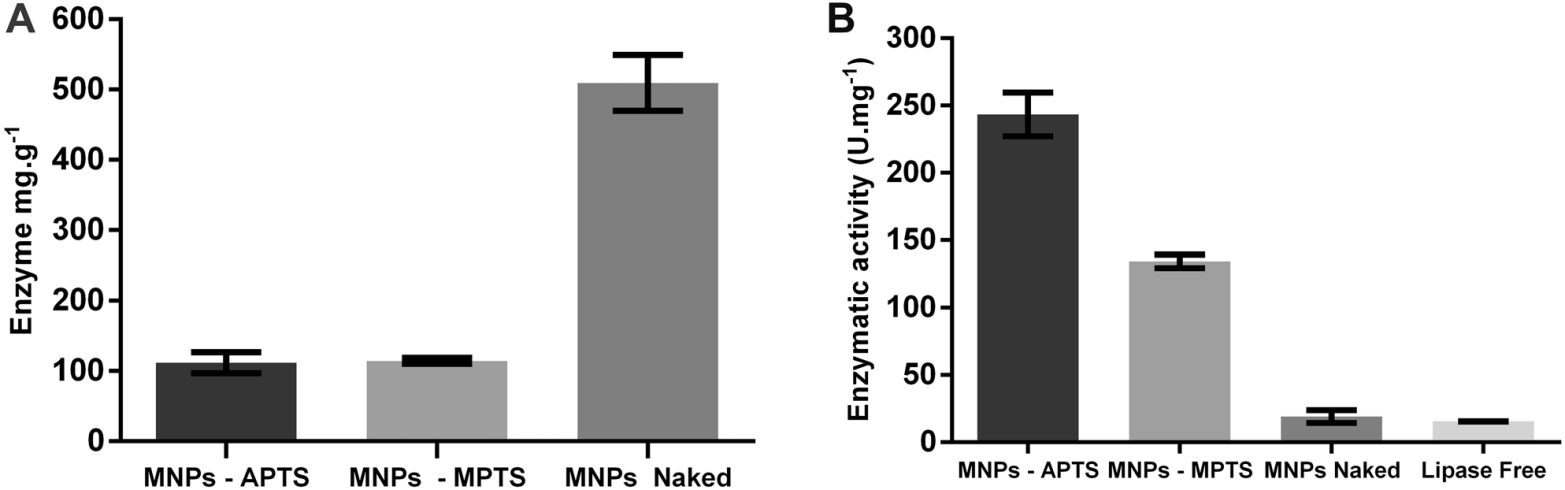
Quantification of conjugated enzymes (**A**), enzymatic activity of immobilized enzymes (**B**).

The results showed that the immobilization strategy strongly influenced surface loading and that the physical adsorption conjugation method was more efficient when compared with the covalent coupling strategies. A loading of 111.69 mg g^−1^ was found for covalent coupling with APTS, 114.43 mg g^−1^ for covalent coupling with MPTS, and 509.48 mg g^−1^ for immobilization by physical adsorption (Fig. 5 and Table 1). There was no significant difference regarding surface loading in the tests using the covalent coupling strategy. This observation suggests that, although there is an evident influence of surfaces with different chemical natures, the effect of the free organic group on conjugation appears to be small, concerning surface loading under the same experimental conditions.

**Table 1:** Surface loading and catalytic activity values for the nanomagnetic supports evaluated.

Support	Dosage (mg g^−1^)	Activity (U mg^−1^)
MNPs-naked	509.48 ± 30.21	21.21 ± 3.66
MNPs-APTS	111.69 ± 10.62	256.89 ± 21.62
MNPs-MPTS	114.43 ± 2.94	133.96 ± 3.41

In a previous study, Wang et al.^55^ determined that 0.13 mg mg^−1^ is the approximate theoretical value for surface loading of lipases on 14 nm spherical and monodisperse nanoparticles. Our covalent coupling results show a good correlation with these values. However, the surface loading for physical adsorption is 4.5 times higher than expected, indicating that this immobilization strategy possibly involves the formation of multilayers.

Catalytic activity assays (Table 1) demonstrated that immobilization by covalent coupling with APTS had the highest activity with 256.89 ± 21.62 U mg^−1^, followed by MNPs-MPTS with 133.96 ± 3.41 U mg^−1^ and physical adsorption with 21.21 ± 3.66 U mg^−1^ (Fig. 5B and Table 1). These data corroborate the hypothesis of the formation of multilayers in the loading of lipases by physical adsorption, given that surface loading can interfere with catalytic activity. A higher lipase load is thought to turn the enzyme into an intermolecular steric obstacle, which restricts substrate and product diffusion^55^.

Catalytic activity is also strongly influenced by the orientation of the enzyme on the immobilization supports. The active site of the enzyme may be randomly involved with the conjugation process by physical adsorption or even by covalent coupling. As the surface of the magnetic supports is charged, physical adsorption is carried out mainly through electrostatic attraction. The zeta potential indicated that the surface of the magnetic nanoparticles is negative (− 75 mV). Therefore, it is expected that immobilization by physical adsorption involves the more hydrophilic and positive side of the enzyme. The polypeptide “cap” of the lipase has been reported to be primarily hydrophobic towards the catalytic “pocket” and hydrophilic on its outer surface^56^. One hypothesis would be that this hydrophilic region would be involved in the immobilization process, significantly reducing the catalytic activity of lipases on the surface by not opening the lid of the active site. For this reason, some studies have reported a gain in activity with hydrophobic supports^57–59^.

The MPTS ligand strategy promotes covalent bonding through a disulfide bridge between the SH of the ligand and the SH of cysteine and methionine residues present in the enzyme structure. In fact, lipases are rich in cysteine and form disulfide bonds to maintain their structure, contributing to conformational stability.

A study by Bordes et al.^60^ that included a primary sequence alignment confirmed the presence of four disulfide bridges (Cys30–Cys299, Cys43–Cys47, Cys120–Cys123, Cys265–Cys273) in the lipase of *Yarrowia lipolytica*. The catalytic activity was higher with the SH ligand as a support than with physical adsorption, although lower than that observed when immobilization was achieved with the APTS ligand. The formation of covalent bonds through disulfide bridges with amino acids that have sulfur in their structure may have reduced the conformational flexibility and affected the activity of the lipase. On the other hand, the disulfide bond between the Cys265–Cys273 amino acids is relatively close to the lipase active site, and the possible binding of the SH ligand with these amino acids may have partially compromised the catalytic activity. In the in silico study by Bordes^60^, a free cysteine (Cys-244) close to the lipase active site was also reported.

**Figure 6:**
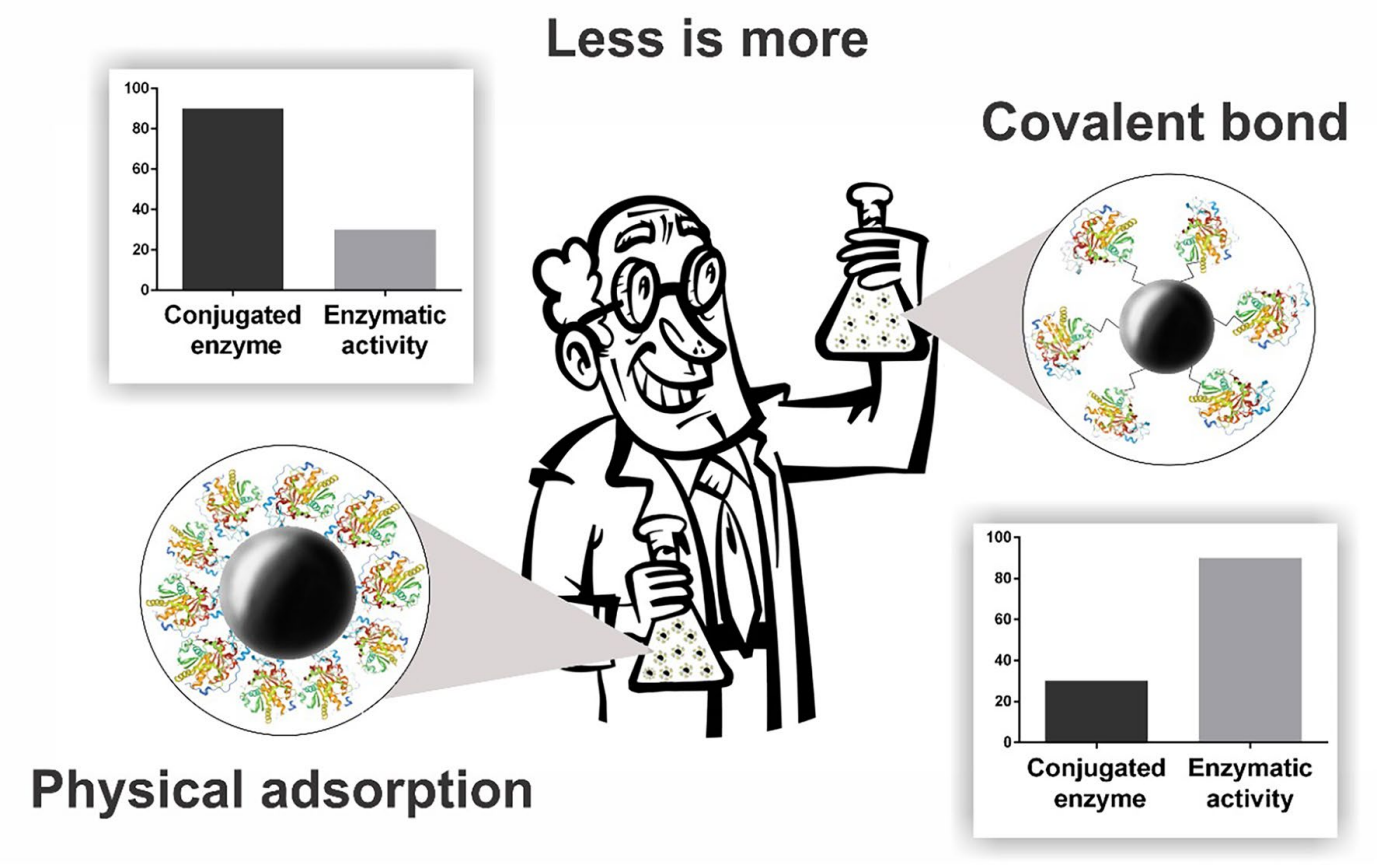
Schematic summary of the immobilization strategies (physical adsorption and covalent coupling).

In the covalent coupling with the APTS ligand, an imine bond (C=N) is formed between the amino group of the ligand and the carbonyl group of the enzyme. This immobilization strategy showed more significant catalytic activity because imine binding probably occurs at sites distant from the lipase active site. An in silico study by Zivkovic et al.^61^ on a lipase from *Candida rugosa* showed that 63% of the lipase surface was nonpolar. An analysis of the distribution of ionizable groups of amino acids in polar areas of this lipase showed that they dominate the molecule's surface in the region away from the active site. In these circumstances, the use of the APTS ligand may have yielded higher activity values by favoring the covalent bonding of the ligand's amine group with the carbonyl group at sites distant from the enzyme active site. At pH ~ 7, an overall negative charge predominates, as acidic amino acids are more numerous than basic amino acids^61^. Figure 6 presents a summary of the immobilization strategies (physical adsorption and covalent coupling). The physical adsorption binding strategy had higher surface loading and low catalytic activity. On the other hand, covalent coupling had an excellent catalytic activity with very low surface loading.

### Stability assessment (pH and temperature)

**Figure 7:**
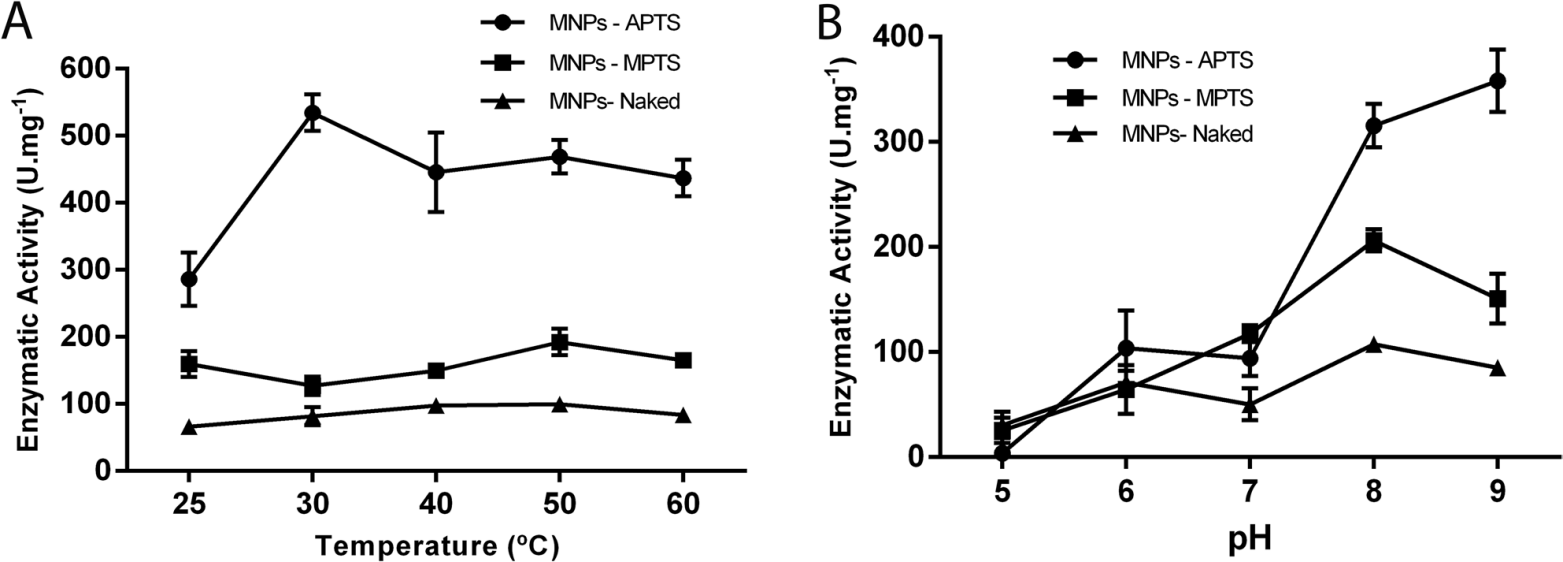
Evaluation of the stability of lipases immobilized by different conjugation strategies. Effect of temperature (**A**) and effect of pH (**B**).

The stability of enzymes (temperature and pH) is an essential parameter for different applications in biocatalysis. In this work, we observed that the immobilization processes evaluated improved thermal stability in relation to free lipase (Fig. 4D). Furthermore, it was possible to check that the nanoconjugates preserve their catalytic activity throughout the range evaluated in this work (Fig. 7A). The nanomagnetic supports appear to have a protective effect at high temperatures at which the enzyme can be deactivated. It has been reported that this improvement may be due to reduced enzymatic movement and structural changes attributed to its attachment to the support, which helps maintain a stable enzymatic conformation and prevents impairment of enzymatic function^62–64^.

Regarding pH, the highest activity values for all systems evaluated were in the range of pH 8 and pH 9. All immobilization strategies showed better stability at alkaline pH (Fig. 7B). The isoelectric pH of lipases is generally between 4.5 and 5.5, which may explain the decrease in activity at acidic pH. It has been reported that protein aggregation near isoelectric pH may be one of the reasons for imprecise enzyme conformation and inhibition of lipase activity^65^. Another possibility may be related to the net potential of the support, where the optimal pH of the immobilized enzyme shifts to more alkaline values. This shift is due to the change in the degree of ionization of the enzyme's active sites^66^.

### Reuse of immobilized lipase (recyclability)

**Figure 8:**
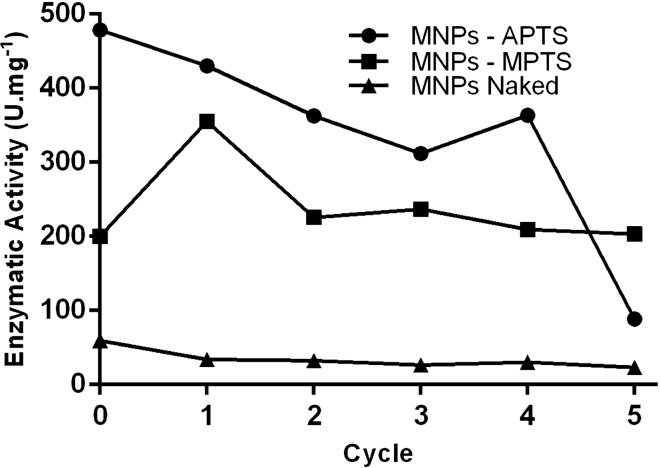
Residual enzymatic activity after five cycles of reuse of immobilized enzymes.

The separation and recycling of enzymes from the reaction medium directly impact economic viability and are one of the main bottlenecks in biocatalysis. In addition to stability and increased catalytic activity, magnetic supports allow the simple separation, recovery, and recycling of lipases using an external magnetic field. To investigate reusability, lipases immobilized on magnetic nanoparticles were separated by an external magnetic field, washed with PBS after each cycle, and redispersed in a p-NPP solution for the next hydrolysis reaction cycle. In the physical adsorption immobilization strategy, the conjugated lipase retained 23,142 U mg^−1^ of catalytic activity after five cycles (Fig. 8). In the covalent coupling strategy using the MPTS ligand, the enzyme maintained 100% of catalytic activity after five cycles (202,929 U mg^−1^). Lipase immobilized with the APTS ligand had a considerable decrease in activity with cycle progression. The fourth cycle retained 363,388 U mg^−1^ of activity and only 88,653 U mg^−1^ remained after the fifth hydrolysis cycle.

In general, decreased activity can result from denaturation, loss of stability, diffusional limitations, and detachment of lipase molecules from the surface of the magnetic support. The decrease in activity in the physical adsorption immobilization strategy seems to be related to the high surface loading. The release of lipases would then occur due to shear forces during agitation. For this reason, the immobilization strategy by physical adsorption is considered a method with low reuse potential. The decrease in catalytic activity of lipases immobilized with APTS ligand along the recycling cycles may be related to mass transfer restrictions due to the amount of product on the matrix surface^67^. In the immobilization using MPTS, the strong covalent bond mediated by the thiol group appears to delay enzyme release, making it reusable for more cycles. These results agree with previously reported observations on covalently immobilized enzymes^68–70^.

Our results indicate that the immobilization of lipases with MPTS, although resulting in lower catalytic activity than that of MNPs-APTS, can be a promising alternative for reusing enzymes after several recycling cycles. These results provide new insights into the biocatalysis industry and may help scale up various applications involving immobilized enzymes. However, studies involving substrate diffusion over immobilized enzymes, the possibility of contamination of the final product, and the environmental impact of nanomagnetic supports are still necessary for full-scale applications. A cost analysis considering all the points discussed in this work is fundamental, in addition, such as to the costs of materials and energy for scaling the process. The correct selection of the immobilization strategy on the nanomagnetic supports is determinant in the catalytic process's reaction yield and economics.

## Conclusions

This study evidenced the differences between three strategies of enzyme immobilization on nanomagnetic supports using *Rhizomucor miehei* lipase as a study model. The results showed that, although the physical adsorption immobilization strategy was more efficient (509.48 mg g^−1^), it resulted in lower catalytic activity (21.21 U mg^−1^). The covalent coupling strategy using the APTS ligand showed a lower yield of surface charge (111.69 mg g^−1^) but higher catalytic activity (256.89 U mg^−1^), with retention of 75.76% of the activity in the fourth reuse cycle. MPTS-mediated immobilization through a free SH ligand showed a surface charge of 114.43 mg g^−1^ and catalytic activity of 133.96 U mg^−1^. This covalent coupling strategy retained 100% of the activity after five hydrolysis cycles. These results show that the highest number of immobilized enzymes is not always the best way to decide on a conjugation strategy. Our findings are essential for the development of nanoconjugates with high enzymatic activity, which can guarantee the success of several industrial applications.

## Supplementary Information

Supplementary Information.

## Data Availability

All data generated or analysed during this study are included in this published article and its supplementary information files.
