# Supplementary material for: Impact of immobilization strategies on the activity and recyclability of lipases in nanomagnetic supports
Source: Sci Rep. 2022 Apr 26;12:6815. doi: 10.1038/s41598-022-10721-y (PMC9042828; doi:10.1038/s41598-022-10721-y)
Supplement: Supplementary file 1 — Supplementary Information. [file 41598_2022_10721_MOESM1_ESM.docx]

***Supporting Information***

**Less is more: impact of immobilization strategies on the activity and recyclability of lipases in nanomagnetic supports**

Thais de Andrade Silva^1^, Wanderson Juvêncio Keijok^1^, Marco Cesar Cunegundes Guimarães^1^, Sérvio Túlio Alves Cassini^2^ and Jairo Pinto de Oliveira^1*^

^1^Federal University of Espírito Santo, Av Marechal Campos 1468, Vitória, ES 29.040-090, Brazil

^2^Federal University of Espírito Santo, Av Fernando Ferrari 514, Vitoria ES, 29075-910, Brazil

*Corresponding author: jairo.oliveira@ufes.br

**Data availability**


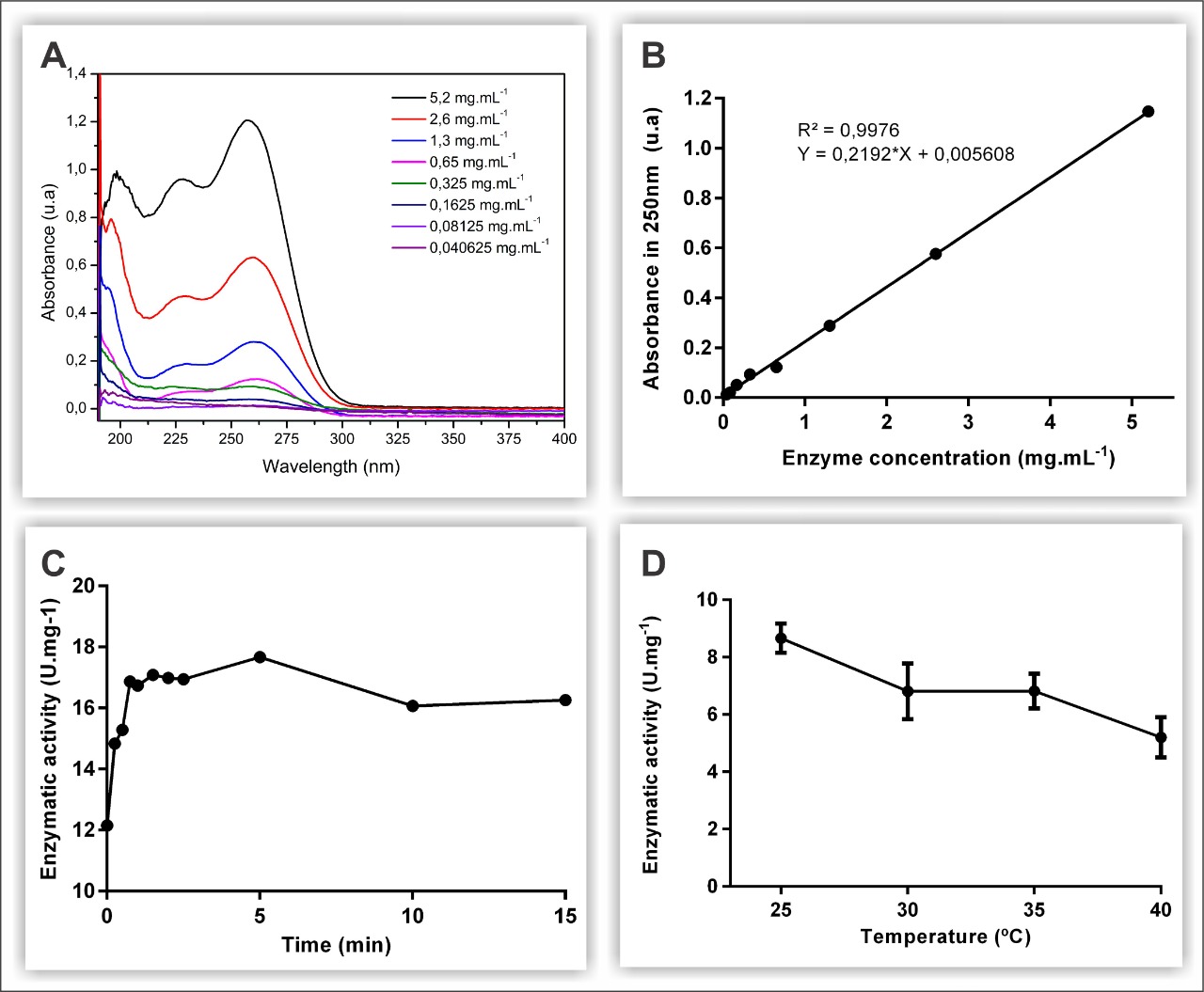


**Figure S1.** UV-Vis Spectrum (A) and Standard Curve of Lipase Concentration (B). Lipase Activity as a function of Time (C) and Temperature (D).


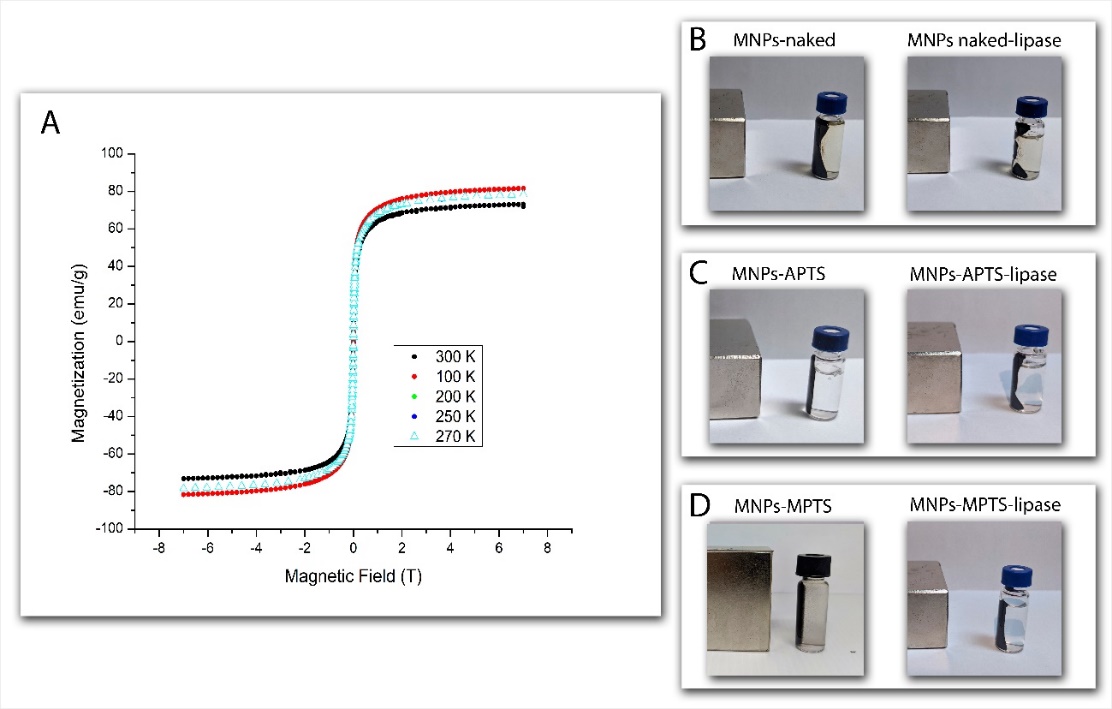


**Figure S2** - Magnetization curve of magnetic nanoparticles at different temperatures (A); Images showing magnetization before and after lipase conjugation for naked MNPs (B); MNPs-APTS (C) and MNPs-MPTS (D).

**
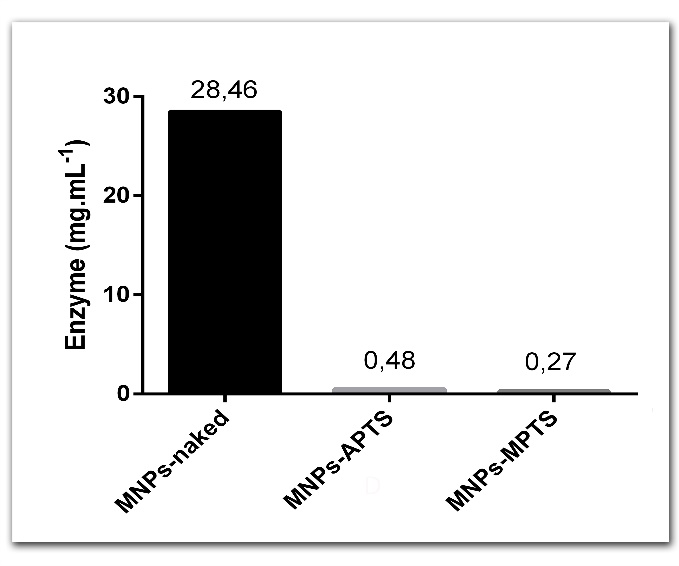
**

**Figure S3.** Desorption assay performed with 250mM NaCl solution.

**Table S1.** Standard Curve of Lipase Concentration

| **Enzyme**  **mg/mL** | **Absorbance in 250m (a.u)** | | |
| --- | --- | --- | --- |
|  | **R1** | **R2** | **R3** |
| 5,200 | 1,127 | 1,146 | 1,169 |
| 2,600 | 0,566 | 0,56 | 0,603 |
| 1,300 | - | 0,275 | 0,303 |
| 0,650 | 0,101 | 0,134 | 0,133 |
| 0,325 | 0,088 | 0,093 | 0,099 |
| 0,1625 | 0,037 | 0,066 | 0,051 |
| 0,0812 | 0,011 | 0,045 | 0,007 |

**Table S2.** Lipase Activity as a function of time.

| **Time (min)** | **Enzimatic Activity**  **(U.mg^-1^)** |
| --- | --- |
| 0,00 | 12,153 |
| 0,25 | 14,840 |
| 0,50 | 15,287 |
| 0,75 | 16,873 |
| 1,00 | 16,740 |
| 1,50 | 17,087 |
| 2,00 | 16,987 |
| 2,50 | 16,946 |
| 5,00 | 17,667 |
| 10,00 | 16,067 |
| 15,00 | 16,260 |

**Table S3.** Lipase Activity as a function of Temperature.

| **Temperature (ºC)** | **Enzymatic Activity**  **(U.mg^-1^)** | | |
| --- | --- | --- | --- |
|  | **R1** | **R2** |  |
| 25 | 8,302 | 9,022 |  |
| 30 | 7,493 | 6,124 |  |
| 35 | 7,244 | 6,391 |  |
| 40 | 5,236 | 5,884 |  |

**Table S4.**  Surface loading and catalytic activity values for the nanomagnetic supports evaluated.

| **Nanomagnetic supports** | **Dosage**  **(mg.g^-1^)** | **Enzymatic Activity (U.mg^-1^)** |
| --- | --- | --- |
| MNPs Naked | 525,379 | 22,567 |
|  | 464,173 | 21,211 |
|  | 538,902 | 13,659 |
| MNPs - APTS | 122,316 | 262,300 |
|  | 101,071 | 210,952 |
|  | - | 256,888 |
| MNPs - MPTS | 117,372 | 133,961 |
|  | - | 139,449 |
|  | 111,486 | 129,601 |
|  | - | 15,400 |
| Lipase Free | - | 15,513 |
|  | - | 15,720 |

**Table S5.**  Catalytic activity data obtained for pH stability assay.

| **Nanomagnetic supports** | **Enzymatic Activity (U.mg^-1^)** | | | | |
| --- | --- | --- | --- | --- | --- |
|  | **pH 5** | **pH 6** | **pH 7** | **pH 8** | **pH 9** |
| MNPs Naked | 45,055 | 60,863 | 67,631 | 95,988 | 79,241 |
|  | 23,515 | 82,501 | 39,670 | 107,351 | 88,331 |
|  | 23,417 | 70,349 | 43,573 | 107,351 | 88,331 |
| MNPs - APTS | 1,750 | 35,109 | 50,859 | 134,203 | 171,718 |
|  | 0,00 | 57,531 | 37,297 | 147,328 | 162,203 |
|  |  | 14,875 | 37,953 | 173,687 | 145,468 |
| MNPs - MPTS | 39,076 | 56,416 | 114,339 | 214,104 | 128,035 |
|  | 21,360 | 46,364 | 111,701 | 210,209 | 148,767 |
|  | 16,209 | 90,341 | 126,653 | 194,377 | 175,279 |

**Table S6.**  Catalytic activity data obtained for temperature stability assay.

| **Nanomagnetic supports** | **Enzymatic Activity (U.mg^-1^)** | | | | |
| --- | --- | --- | --- | --- | --- |
|  | **25 ºC** | **30 ºC** | **40 ºC** | **50 ºC** | **60 ºC** |
| MNPs Naked | 63,235 | 94,654 | 98,162 | 103,991 | 91,097 |
|  | 65,063 | 67,829 | 93,370 | 100,830 | 81,859 |
|  | 71,090 | 83,094 | 101,867 | 94,210 | 78,599 |
| MNPs - APTS | 416,522 | 556,752 | 403,774 | 468,986 | 467,270 |
|  | 257,905 | 504,043 | 487,618 | 493,502 | 414,316 |
|  | 314,292 | 542,533 | 506,250 | 443,735 | 429,270 |
| MNPs - MPTS | 137,584 | 115,219 | 145,500 | 215,109 | 167,237 |
|  | 168,494 | 141,856 | 162,337 | 182,692 | 170,504 |
|  | 173,017 | 124,014 | 141,354 | 179,551 | 157,688 |

**Table S7**. Residual enzymatic activity after five cycles of reuse of immobilized enzymes.

| Cycle | MNPs - APTS | MNPs - MPTS | MNPs Naked |
| --- | --- | --- | --- |
| 0 | 478,373 | 200,305 | 59,230 |
| 1 | 430,097 | 355,087 | 33,851 |
| 2 | 362,803 | 225,768 | 32,287 |
| 3 | 311,893 | 236,879 | 26,606 |
| 4 | 363,388 | 209,410 | 30,387 |
| 5 | 88,653 | 202,929 | 23,142 |
